# Changes in Honey Bee Head Proteome in Response to Dietary 24-Methylenecholesterol

**DOI:** 10.3390/insects11110743

**Published:** 2020-10-29

**Authors:** Priyadarshini Chakrabarti, Ramesh R. Sagili

**Affiliations:** Department of Horticulture, Oregon State University, Corvallis, OR 97331, USA

**Keywords:** nurse bee, honey bee proteomics, phytosterol, 24-methylenecholesterol, honey bee nutrition, honey bee physiology

## Abstract

**Simple Summary:**

Phytosterols are important micronutrients that are essential for production of insect molting hormones and cellular membrane integrity. Past research has shown that the key phytosterol that honey bees need is 24-methylenecholesterol. This phytosterol improves honey bee longevity and sustains brood production. Hence, it is important to understand how 24-methylenecholesterol can shape honey bee physiology by altering protein profiles of vital honey bee tissues. Nurse bees secrete glandular secretions (brood food) using hypopharyngeal and mandibular glands in their head regions. Further, it has been shown that this sterol is selectively accumulated in nurse bee heads. Thus, it is imperative to examine the protein profiles of nurse bee heads, in response to dietary 24-methylenecholesterol manipulation. In this study, groups of newly emerged nurse bees were fed with varying concentrations of dietary 24-methylenecholesterol, while the control groups received no sterol. We found that dietary sterol manipulation altered the protein profiles in nurse bee heads, with important nutritional marker proteins being upregulated in high dietary sterol groups. The important proteins identified in this study may serve as vital markers of nutritional stress related to sterols in honey bees, paving the way for future research on bee nutrition.

**Abstract:**

Phytosterols are important micronutrients that are precursors of important molting hormones and help maintain cellular membrane integrity in insects including bees. Previous research has shown that 24-methylenecholesterol is a key phytosterol that enhances honey bee longevity and improves nurse bee physiology. Nurse bees have the ability to selectively transfer this sterol to developing larvae through brood food. This study examines the physiological impacts of 24-methylenecholesterol on nurse bees, by analyzing the protein profiles of nurse bee heads upon dietary sterol manipulation. Dietary experimental groups consisting of newly emerged honey bees were provided with varying concentrations of 24-methylenecholesterol for three weeks. At the end of the study, honey bees were collected and proteomic analysis was performed on honey bee heads. A total of 1715 proteins were identified across experimental groups. The mean relative abundances of nutritional marker proteins (*viz*. major royal jelly proteins 1, 4, 5, 7) were higher in experimental groups supplemented with higher dietary sterol concentrations, when compared with the control dietary group. The mean relative abundances of important enzymatic proteins (aminopeptidase and calcium-transporting ATPase) were higher in control groups, whereas mean relative abundances of oxysterol-binding protein and fatty acid-binding protein were higher in higher dietary sterol groups.

## 1. Introduction

Nutrition is the key to honey bee colony health and survival, ensuring individual bees are healthy to counteract detrimental impacts of pathogens, parasites and pesticides [1,2,3,4]. Yet, poor nutrition is cited as one of the important factors for global bee declines. For bees, pollen and nectar are the two important sources of nutrition, with pollen providing vital macronutrients (viz. proteins and carbohydrates) [2] and micronutrients such as phytosterols [5,6]. Along with macronutrients, micronutrients are also equally important for optimal nutrition and sustenance of honey bees. Of these micronutrients, phytosterols play a critical role as precursors of insect molting hormones and assist in maintaining insect cellular membrane integrity [7,8]. Since like all insects, bees are sterol auxotrophs [8,9], they are dependent on pollen for their vital phytosterol needs.

Each bee species has a different phytosterol requirement. Bumble bee larval development has been found to benefit positively with 24-methylenecholesterol, β-sitosterol and δ5-avenasterol (also called isofucosterol) [10]. Stingless bees on the other hand exhibited campesterol, stigmasterol, sitosterol and δ5-avenasterol accumulations in their cephalic glands [11]. Previous studies have documented that in honey bees 24-methylenecholesterol is transferred in maximum amounts selectively by the nurse bees to the developing brood via brood food [12,13], followed in decreasing order by sitosterol, δ5-avenasterol and campesterol. Honey bees have been shown to perform well (increased brood production and enhanced longevity of workers) when supplemented with 24-methylenecholesterol [14]. Previous studies have also shown that radiolabeled 24-methylenecholesterol from diets was selectively taken up by nurses, transferred to the brood, retained in growing brood and later on transferred to the new queens in the progeny [12]. Further, Feldlaufer, 1986 [15] reported that 24-methylenecholesterol accounted for 50% of the sterols found in honey bee pupae (13 days post-oviposition). Recent studies have shown that this particular sterol may act as a phagostimulant and higher consumption of artificial diets laced with 24-methylenecholesterol improves honey bee longevity, increases head protein and abdominal lipid contents, and the sterol is progressively assimilated across various tissues in honey bees [6,16]. Hence, 24-methylenecholesterol is a key phytosterol for colony growth and longevity in honey bees.

Nurse bees are vital to the functioning of the hive, as they selectively take up sterols from dietary sources [12] and transfer it to the growing larvae through brood food. The brood food consists of proteinaceous secretions from the nurse bee brood food glands (viz. hypopharyngeal and mandibular glands) located in the heads [17,18,19]. Recently, a number of studies have focused on gaining insights on transcriptomic and proteomic changes in the honey bee heads in response to a stressor (nutrition or pesticides) [20,21,22,23,24]. Since honey bee heads house important brood food-producing glands, sensory structures (antennae), the brain and organs for visual acuity and taste, it is important to understand the impacts of dietary manipulations on the head regions of the nurse bees. Further, as little is understood about the physiological impacts of 24-methylenecholesterol (a key phytosterol) on honey bees, it is also important to understand how this specific phytosterol regulates nurse bee physiology and overall bee health.

This study is part of a larger study conducted in 2017, where newly emerged nurse bees were fed with synthetic diets laced with varying concentrations of 24-methylenecholesterol [6]. For this study, live honey bees were collected at the end of the experiment and head proteome was analyzed in response to the dietary manipulation of this important sterol. We identified nurse bee head proteins in sterol-supplemented dietary groups and controls, classified the identified proteins into relevant groups, and assessed the impacts of dietary 24-methylenecholesterol limitations on the relative abundances of these identified proteins.

## 2. Materials and Methods

### 2.1. Experimental Design and Dietary Supplementation

Three frames of ready-to-emerge honey bees (*Apis mellifera* L.) were collected from each of the six sister-queen colonies (to mitigate genetic variation), and all 18 frames were placed in an incubator overnight at 33 °C and 55% RH (Relative Humidity). All newly emerged bees were thoroughly mixed, and from that pool, 170 newly emerged honey bees were randomly allocated to each replicate cage of the experimental groups (control and five dietary treatments). Hence, we had a total of 3060 newly emerged bees (6 experimental groups * 3 replicate cages * 170 honey bees in each cage) in this study. The diet formulation is described in detail in our published study [6]. Briefly, 2 g of the synthetic diet contained 810 mg of amino acid powder (Nutricia, Zoetermeer, Netherlands), 1.171 g sucrose (C&H sugar, Crockett, CA, USA), 17 mg Wesson’s salt (MP Biochemicals, Irvine, CA, USA), 2 mg of zinc gluconate (Millipore Sigma, Burlington, MA, USA), 4 µL of B-vitamin mixture (Durvet) and 450 µL of 40% sucrose syrup. An equal volume of acetone solution was added to the dry diet mixtures to create treatment diets with the following concentrations of synthetic 24-methylenecholesterol (as percentage dry diet weight): 0.1% (treatment group S1), 0.25% (treatment group S2), 0.5% (treatment group S3), 0.75% (treatment group S4) and 1.0% (treatment group S5). The synthetic 24-methylenecholesterol was obtained from Expert Synthesis Solutions (London, ON, Canada). The control diet group received an equal volume of acetone, without any 24-methylenecholesterol. There were three replicate cages for each control and sterol diet treatment group. The synthetic diets were replaced weekly. The duration of the study was three weeks. The cages remained in the incubator (33 °C and 55% RH) throughout the study duration and were also provided with 40% sugar syrup and water.

### 2.2. Proteomics of Honey Bee Heads

Live honey bee samples were collected at the end of three weeks for conducting proteomic analysis (this study) and various physiological analyses (results previously published [6]). Proteomics was conducted based on previously established methods [25].

#### 2.2.1. Sample Preparation

Proteomic sample preparations and analyses were performed using the ProteaseMax reagent and protocols (Promega, Madison, WI, USA). At the end of three weeks, for each replicate of the experimental groups (control and S1–S5), the heads of five honey bees were pooled. Thus, a total of 15 honey bee heads were analyzed from each experimental group (five from each replicate). The pooled samples of each replicate cage were homogenized in 2 mL of 50 mM ammonium bicarbonate buffer with 0.04% ProteaseMax reagent and one 3-mm tungsten carbide bead (Qiagen, USA), using a Tissue Lyser II (Qiagen, Germantown, MD, USA; two rounds of 1.5 min at 30 oscillations s^−1^). Homogenized samples were then centrifuged at 20,000× *g* for 30 min at 4 °C (Eppendorf model 5430R, Eppendorf, Enfield, CT, USA) to pellet the debris. Next, a BCA assay (Pierce Biotech BCA Assay Kit, Thermo Scientific, Waltham, MA, USA) was used to quantify protein concentration of the supernatants by measuring absorbance at 562 nm on a BioTek Synergy 2 plate reader (BioTek Instruments, Winooski, VT, USA). The remaining volume of supernatant was submitted to the Mass Spectrometry Center at Oregon State University where nano LC-MS was performed.

#### 2.2.2. Mass Spectrometry

Protein samples were digested by mass-spectrometry grade trypsin (Promega, Madison, WI, USA). Following five minutes of desalting at a flow rate of 5 µL min^−1^, 0.1 µg of the peptide mixtures were loaded on a nanoAcquity UPLC 2 G Trap Column (180 µm × 20 mm, 5 µm). A nanoAcquity UPLC Peptide BEH C18 column (100 µm × 100 mm, 1.7 µm) was used for peptides separation over a 120 min gradient at a flow rate of 500 nL min^−1^. Specifically, mobile phase B was increased from 3% to 10% in first 3 min, then up to 30% in 102 min, rapidly up to 90% in 1 min and held for 4 min, finally down to 3% in 1 min and held for 7 min. The spray voltage and the ion transfer tube temperature were 2400 V and 300 °C respectively. Trypsin digested sample preparations and analyses were performed using established protocols [25,26]. Peptide samples were separated by a Waters nanoAcquity UPLC system (Waters, Taunton, MA, USA) and analyzed by an Orbitrap Fusion Lumos system with nano-ESI source (Thermo Scientific, Waltham, MA, USA). MS data were collected in the Orbitrap analyzer at 120 K resolution (m/z 200) under the positive ion mode. MS/MS spectra were recorded in the linear trap quadrupole analyzer under collision induced dissociation (CID) fragmentation mode with top speed method. The automatic gain control target was set to 4.0 × 105 and 4.0 × 104 for precursor ions and product ions respectively. The mass tolerances values were set at ±10 ppm and 0.6 Da for precursor ions and fragment ions respectively. A maximum of two missed cleavage sites was allowed. Only proteins with high confidence and an overall false discovery rate (FDR) < 1% were considered for analyses.

### 2.3. Data Analysis

All raw data files were initially analyzed with Thermo Scientific^TM^ Proteome Discoverer^TM^ 2.2 software (Thermo Scientific, Waltham, MA, USA) and searched using Sequest HT engine against Uniprot *Apis mellifera* L. protein databases to identify proteins. This software also generated fold change data with adjusted p-values for every treatment group (S1–S5) when compared with control (C). Protein–protein interactions were mapped and the pathways visualized and analyzed by STRING v9.1 [27], PANTHER [28] and Cytoscape v3.6.0 [29] using *Apis mellifera* KEGG database and NCBI taxonomy id: 7460 for *Apis mellifera*. All data were log-transformed in MetaboAnalyst and the datasets were then analyzed by MetaboAnalyst v4.0 [5] for heat maps, principal component analysis (PCA) plots, partial least squares-discriminant analysis (PLS-DA) plots, ANOVA and multiple testing using significant analysis of microarray (SAM) feature with FDR ≤ 0.05 based on Benjamini and Hochberg corrections [30].

## 3. Results

### 3.1. Proteins Identified from Proteomic Analysis

**Figure 1:**
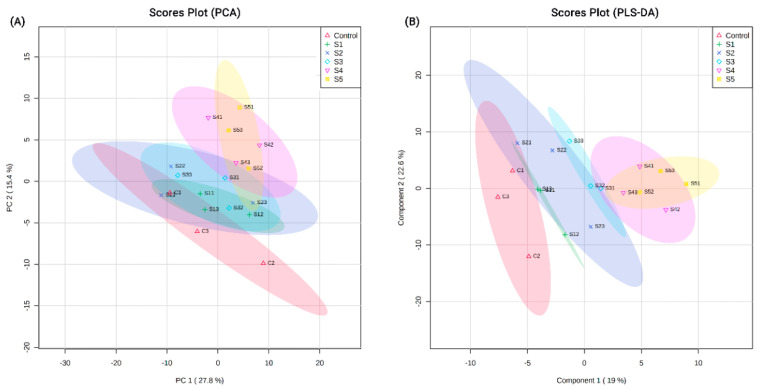
(**A**) Principal component analysis (PCA) plots and (**B**) partial least squares-discriminant analysis (PLS-DA) plots generated by MetaboAnalyst showing the clustering of 1715 detected proteins across the different experimental groups. C indicates control. S1–S5 indicate dietary groups treated with 0.1%, 0.25%, 0.5%, 0.75% and 1.0% dry diet weight of 24-methylenecholesterol respectively. Numbers 1–3 for every group indicate the replicate cage number.

**Figure 2:**
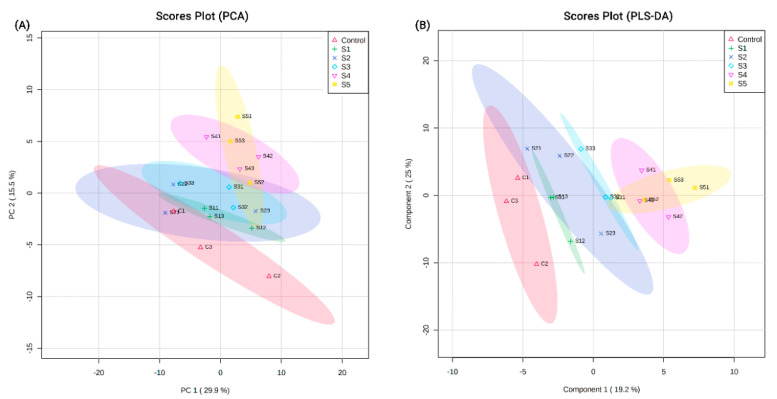
(**A**) PCA plots and (**B**) PLS-DA plots generated by MetaboAnalyst showing the clustering of the 1089 identified proteins across the different experimental groups. C indicates control. S1–S5 indicate dietary groups treated with 0.1%, 0.25%, 0.5%, 0.75% and 1.0% dry diet weight of 24-methylenecholesterol respectively. Numbers 1–3 for every group indicate the replicate cage number.

Proteomic analysis identified a total of 1715 protein groups with high confidence across the experimental groups (control and treatment) (Table S1). Of these, 626 proteins were uncharacterized, indicating that these were not homologous to proteins in the Uniprot *A. mellifera* database. The PCA plots of all 1715 detected proteins indicated a segregation of the protein groups based on the dietary treatments (Figure 1A). This exclusion was further highlighted in the PLS-DA plots, where clusters separated more distinctly based on the experimental dietary groups (Figure 1B). Similar clustering, but with clearer segregations, was observed when PCA (Figure 2A) and PLS-DA (Figure 2B) plots were constructed for the 1089 identified proteins.

**Figure 3:**
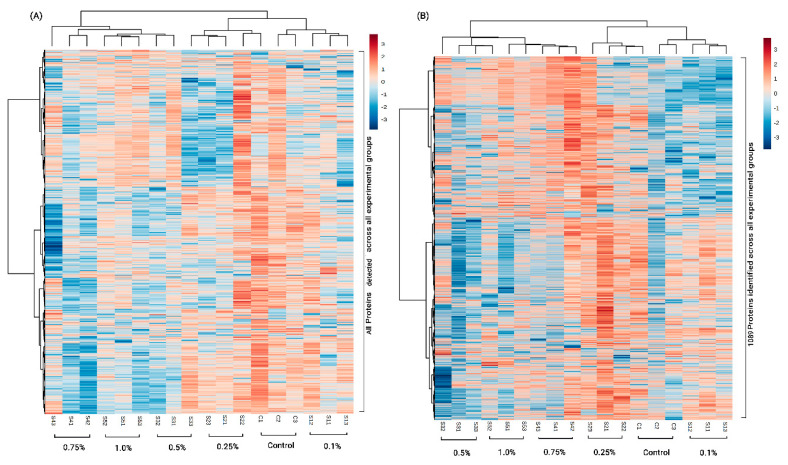
Heat maps plotting the relative abundances of (**A**) all detected 1715 proteins and (**B**) 1089 identified proteins across the treatment groups. C indicates control. S1–S5 indicate dietary groups treated with 0.1%, 0.25%, 0.5%, 0.75% and 1.0% dry diet weight of 24-methylenecholesterol respectively. Numbers 1–3 for every group indicate the replicate cage number.

The heat map for the relative abundances of all detected proteins is shown in Figure 3A, and of the identified 1089 proteins is shown in Figure 3B. The overall clustering pattern observed, as shown in the heat maps, indicates grouping of the proteins based on the dietary treatments, further reiterating the results from the PCA and the PLS-DA analysis. When multiple testing was conducted using the SAM feature, 286 proteins were found to be significantly different (*p* < 0.05) across the treatment groups. Further data analyses were next conducted on the 1089 identified proteins.

**Figure 4:**
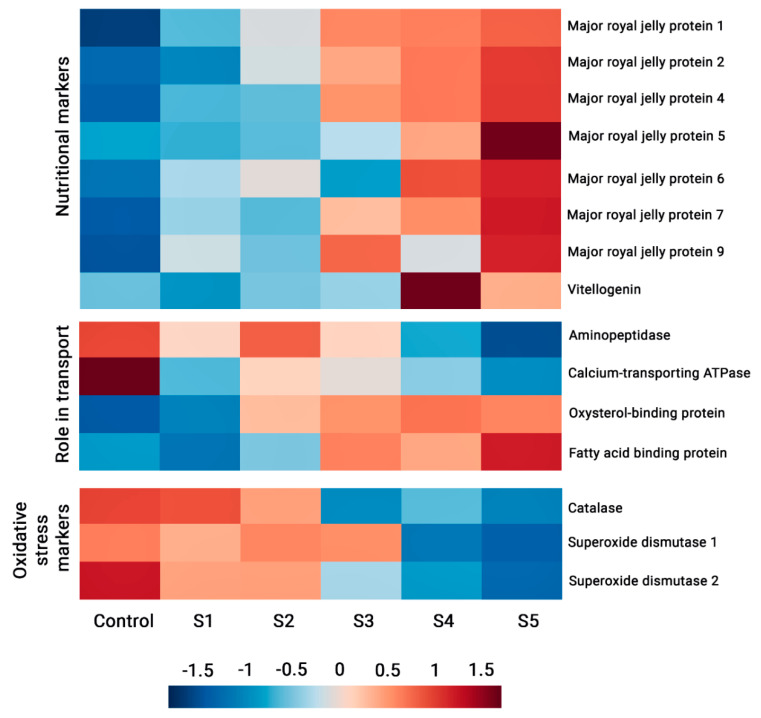
Heat map shows the mean relative abundances of the selected 15 proteins across all treatment groups. S1–S5 indicate dietary groups treated with 0.1%, 0.25%, 0.5%, 0.75% and 1.0% dry diet weight of 24-methylenecholesterol respectively.

Among these 1089 proteins, 15 proteins of interest (chosen for their involvement in nutrition, queen/worker differentiation and division of labor, oxidative stress physiology, lipid movement etc.) were further analyzed. The proteins of interest were: (a) nutritional markers—major royal jelly proteins (MRJPs) 1, 2, 4–7, 9 and vitellogenin; (b) proteins having a role in transport and membrane functions—aminopeptidase, calcium-transporting ATPase, oxysterol-binding protein and fatty acid-binding protein; (c) oxidative stress markers—catalase and superoxide dismutase [Cu-Zn] 1 and 2. Heat maps depicting the mean relative abundances of these 15 proteins are shown in Figure 4. For the MRJPs and vitellogenin proteins, a clear trend is observed in Figure 4, where the mean relative abundances are higher in groups supplemented with higher dietary sterol concentrations, when compared with the control dietary group.

Among the four proteins selected for having a role in transport, a mixed pattern was observed (Figure 4). Mean relative abundances of important enzymatic proteins, for example aminopeptidase and calcium-transporting ATPase, were higher in the control group, and decreased with increasing sterol concentrations in the synthetic diets. On the other hand, honey bees which consumed higher-sterol supplemented synthetic diets exhibited increased mean relative abundances of oxysterol-binding protein and fatty acid-binding protein. The mean relative abundances of the three antioxidant enzymatic proteins—catalase and superoxide dismutase 1 and 2—were the highest in the control group and subsequently decreased in the high sterol-supplemented dietary treatment groups (Figure 4). ANOVA was performed on these above-mentioned 15 proteins using MetaboAnalyst. Among these 15 proteins, the following were found to be significantly different between the experimental groups: MRJPs 1, 4, 5 and 7, superoxide dismutase 1 and 2, catalase, oxysterol-binding protein and fatty acid-binding protein (Table S2). Multiple testing by SAM feature also indicated significant differences in these important proteins between the experimental groups (Table S3).

### 3.2. Protein-Protein Interactions and Functional Enrichments

As mentioned earlier, all raw data files were initially analyzed with Thermo Scientific^TM^ Proteome Discoverer^TM^ 2.2 software and fold change data with adjusted p-values for each treatment group (S1–S5), compared with control (C), were generated next. Only those proteins exhibiting significant fold changes with adjusted *p*-values ≤ 0.05 were selected (Table S4) and further analyzed with Cytoscape. For each pair of experimental groups (S1 and control, S2 and control, S3 and control, S4 and control, S5 and control), proteins that were biologically linked and significantly regulated (downregulated or upregulated as indicated by fold changes) were mapped, and the resultant networks are shown in Figure S1. The protein networks (Figure S1) revealed that the networks for groups S3/control, S4/control and S5/control were similar, when compared with S1/control and S2/control. The names and accession numbers for these proteins in the network can be found in Table S1.

An in-silico protein–protein interaction map was further plotted by STRING (Figure S2), and pathway enrichment analysis was performed by STRING and PANTHER. The results for protein–protein interactions are provided in Table S5, the results for KEGG pathway enrichment are provided in Table S6, and the results for molecular function enrichment are provided in Table S7. Overall, 47 pathways were enriched that include important pathways such as metabolic pathways (carbon metabolism, purine metabolism, cysteine and methionine metabolism, glutathione metabolism etc.), autophagy and degradation pathways, signaling pathways etc. (Table S6). Additionally, six molecular function enrichments found were catalytic activity, purine ribonucleotide binding, purine ribonucleoside triphosphate binding, ion binding, organic cyclic compound binding and heterocyclic compound binding functions (Table S7).

### 3.3. Classification of Proteins Identified

**Figure 5:**
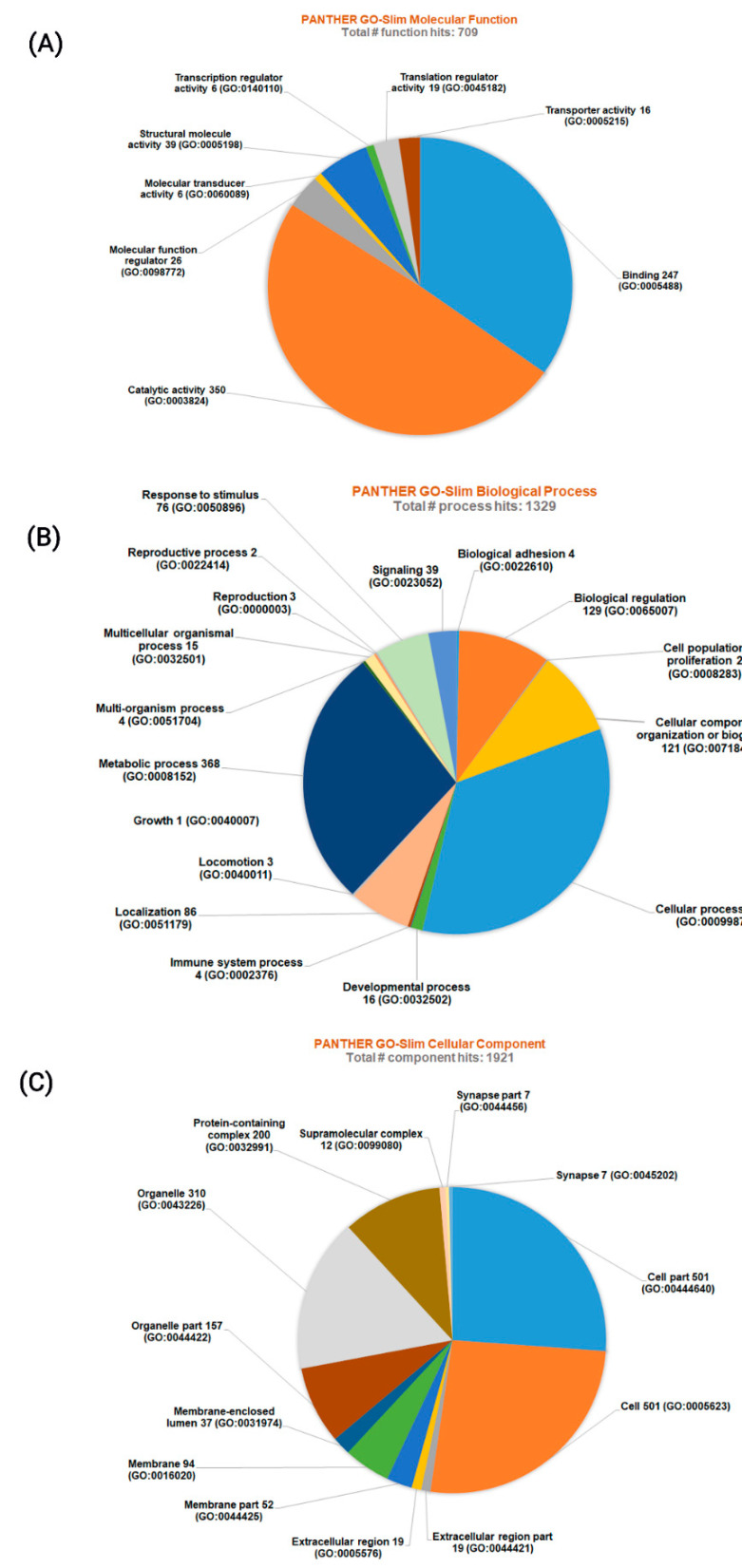
Classification of the 1089 proteins identified across all treatment groups based on the PANTHER GO analysis for (**A**) molecular functions, (**B**) biological processes and (**C**) cellular components. Numbers next to each family/subfamily name indicate the number of function (molecular function), process (biological process) and component (cellular component) hits.

Using PANTHER GO analysis, all 1089 identified proteins were classified into three major groups based on their molecular functions, biological processes and cellular components/localizations (Figure 5).

When proteins were grouped based on their molecular functions, the total number of function hits was 709. Among these, the most abundant were proteins having binding functions and catalytic activity. Other molecular functional groups identified were transcription regulator activity, translation regulator activity, transporter activity, molecular function regulator, molecular transducer activity and transcription regulator activity (Figure 5A).

When proteins were grouped based on their biological processes, the total number of biological process hits was 1329 and proteins having a role in cellular process and metabolic processes were in the majority. The other groups identified were biological adhesion, biological regulation, cell population proliferation, cellular component organization or biogenesis, developmental process, immune system process, growth, localization, locomotion, multi-organism process, multicellular organismal processes, reproduction, reproductive process, response to stimulus and signaling (Figure 5B).

The protein grouping based on their cellular localizations resulted in 1921 total component hits. Among this group, proteins within a cell and in the organelles were predominant. Other groups observed were proteins that were part of extracellular regions, cell membranes and membrane structures, membrane-enclosed lumen, protein-containing complex, within the extracellular matrix, supramolecular complex and synapse (Figure 5C).

## 4. Discussion

To our knowledge this is the first study to report changes in the nurse bee head proteome in response to diets containing different concentrations of a key phytosterol (24-methylenecholesterol). Our findings elucidate the potential role of this sterol in individual bee health and overall colony performance.

Previous studies have shown high quantities of 24-methylenecholesterol in the hypopharyngeal glands of honey bees that consumed an artificial diet containing this particular sterol [13]. This may have significant implications, as hypopharyngeal glands are the sites of MRJP1 production [31], and MRJP1 was more abundant in bees fed sterol-rich diets in our current study. MRJP1 is an important constituent of the proteinaceous glandular secretions of the nurse bee hypopharyngeal and mandibular glands. MRJP1 plays an important role in reproductive maturation of larvae and age polyethism in honey bees [32,33]. MRJP1 has also been recently reported to be a 24-methylenecholesterol carrier [34]. In the current study, bees fed diets with higher concentrations of 24-methylenecholesterol had significantly higher MRJPs (Table S2), including MRJP1. These results further support our previous findings [6,16], where we found higher head proteins and higher assimilation of 24-methylenecholesterol in bees that were fed diets containing high concentrations of 24-methylenecholesterol. This suggests a potential role of this sterol in protein synthesis in the brood food-producing glands, as well as the overall nutritional state of the nurse honey bees.

Honey bees fed diets containing higher concentrations of 24-methylenecholesterol had higher vitellogenin than bees from the control group, though this difference was not significant (Table S2). Vitellogenin is a female-specific glycolipoprotein in insects, synthesized by fat body cells [35,36]. Increased levels of vitellogenin in honey bees pertaining to sterol treatment groups suggests that sterols may improve overall fat body cell function (particularly protein synthesis) or play some role in the production of vitellogenin, specifically. These results are in agreement with our previous findings, where honey bees fed synthetic diets containing higher concentrations of 24-methylenecholesterol, and exhibited significantly higher abdominal fat contents at the end of the experiment [6,16]. In honey bee workers, this protein is linked to onset of foraging and has been demonstrated to influence the collection of pollen vs. nectar [37,38]. It also contributes to the reduction of oxidative stress and positively impacts longevity [38,39]. As such, vitellogenin is among the most important drivers of colony life history, strength and division of labor.

Among the other proteins, fatty acid-binding protein was significantly higher in honey bees from dietary treatment groups laced with higher concentrations of sterols (Table S2). Fatty acid-binding proteins are important lipid chaperones, responsible for lipid traffic through cellular membranes [40,41]. Mean relative abundance of oxysterol-binding proteins increased significantly with increasing dietary sterol (Table S2). These proteins are highly conserved sterol transport proteins that play a role in lipid metabolism [42]. In a field-realistic scenario, colonies with limited sterol intake in their diets can result in lower abundances of these proteins, and as a result, this may affect lipid uptake and transfer within the nurse bee body and may result in downstream cascading effects on the brood. Aminopeptidase and calcium-transporting ATPase, on the other hand, were found in higher abundance in experimental groups with lower sterol concentrations, though this difference was not statistically significant. Both these proteins play a role in membrane structure or function; aminopeptidases are components of cellular membranes and organelles [43], whereas calcium-transporting ATPase proteins transport Ca^+2^ across cell membranes and are vital for maintaining intracellular Ca^+2^ [44]. Superoxide dismutase 1 and 2 and catalase were found in significantly higher abundance in the control group and low sterol treatment groups (Table S2). As these are important antioxidant enzymatic proteins [45], this suggests some degree of oxidative stress in bees from the groups that lacked sterol or had low sterol concentrations. The resultant change in these proteins due to dietary 24-methylenecholesterol limitations can not only alter nurse bee physiology, but also may have an impact on their brood food-producing glands, the nutritional quality of the brood food and on a long term, have an effect on the overall colony growth and function.

It is also noteworthy that the following proteins—fatty acid-binding protein, oxysterol-binding protein, superoxide dismutase 1 and 2 and catalase—were found to be significantly different between groups upon multiple testing (Table S3). We are unable to provide a plausible explanation for the differential regulations of these proteins in sterol-deficient bees, but this observed phenomenon affirms the importance of sterols in membrane stability and function [46] and requires further investigation.

A majority of the significantly enriched pathways (see Results, Section 3.2.) are metabolic pathways, indicating the need for further research on sterol metabolism and the impacts of dietary sterols on other metabolic pathways in honey bees. In our previous study [6], we observed that when bees were supplemented with 0.1–1.0% 24-methylenecholesterol concentrations in their diets (S1–S5 respectively), the highest survival was recorded in the group supplemented with 0.5% sterol in the diets (S3). For consumptions and abdominal lipid content analysis, the S3 group exhibited higher significant differences when compared with control and low sterol groups and no significant difference when compared with the S4 (0.75%) and S5 (1.0%) groups. In the present study, similarities in protein networks (Figure S1) between higher sterol and control groups (S3 and control, S4 and control, S5 and control) suggest that dietary supplementation of 24-methylenecholesterol after a certain concentration may not provide any additional benefits. These results thus support our previous findings [6] that 0.5% concentration of 24-methylenecholesterol (S3) may be an optimal concentration for formulating supplemental protein diets for honey bees. When proteins were identified according to their molecular functions, biological processes and cellular localizations (see Results, Section 3.3.), proteins having binding and catalytic activity, proteins having a role in cellular processes/molecular chaperone locomotion, and intracellularly located proteins were most abundant, respectively. This further reaffirms the need to better understand the role of sterols in protein transport, membrane stability and important metabolic processes in honey bees. In this study, the bees in the experimental cages were three weeks old when proteomics was conducted. These bees might have transitioned to the foraging task if they were in a natural hive environment. Future research thus should also be directed towards understanding honey bee physiological changes due to phytosterol limitations across multiple age cohorts.

## 5. Conclusions

This study reports novel findings on the impacts of dietary manipulations of a key phytosterol (24-methylenecholesterol) on honey bee proteins. The head proteome was significantly altered in nurse bees consuming diets containing varying concentrations of 24-methylenecholesterol. This study elucidates the impacts of sterol limitations on nurse bees, and alludes to the cascading downstream effects on brood food production and brood rearing. Important proteins identified in this study, such as MRJPs and vitellogenin, may serve as important markers of nutritional stress related to sterols in honey bees and pave the way for future investigations into similar impacts across vital honey bee body tissues.

## Supplementary Materials

The following are available online at https://www.mdpi.com/2075-4450/11/11/743/s1, Figure S1: The protein networks as constructed in Cytoscape based on the fold change data generated from Thermo Scientific^TM^ Proteome Discoverer^TM^ 2.2 software, Figure S2: Protein–protein interaction map plotted by STRING, Table S1: Detailed list of all proteins identified in the proteomic study of nurse bee heads, Table S2: The results from the ANOVA tests performed in MetaboAnalyst, Table S3: Results from multiple testing of the proteins across all treatment groups using SAM feature in MetaboAnalyst, Table S4: Fold change data generated from Thermo Scientific^TM^ Proteome Discoverer^TM^ 2.2 software, Table S5: Results from protein–protein interactions as generated by STRING, Table S6: Results from KEGG pathway enrichment analysis, Table S7: Results from molecular function enrichment analysis.

## References

[1] Alaux C., Ducloz F., Crauser D., Le Conte Y. (2010). Diet effects on honeybee immunocompetence. Biol. Lett..

[2] Brodschneider R., Crailsheim K. (2010). Nutrition and health in honey bees. Apidologie.

[3] Alaux C., Dantec C., Parrinello H., Le Conte Y. (2011). Nutrigenomics in honey bees: Digital gene expression analysis of pollen’s nutritive effects on healthy and *Varroa*-parasitized bees. BMC Genom..

[4] Schmehl D.R., Teal P.E.A., Frazier J.L., Grozinger C.M. (2014). Genomic analysis of the interaction between pesticide exposure and nutrition in honey bees (*Apis mellifera*). J. Insect Physiol..

[5] Chakrabarti P., Morre J.T., Lucas H.M., Maier C.S., Sagili R.R. (2019). The omics approach to bee nutritional landscape. Metabolomics.

[6] Chakrabarti P., Lucas H.M., Sagili R.R. (2019). Evaluating effects of a critical micronutrient (24-methylenecholesterol) on honey bee physiology. Ann. Entomol. Soc. Am..

[7] Behmer S.T., Nes W.D. (2003). Insect sterol nutrition and physiology: A global overview. Adv. Insect Phys..

[8] Carvalho M., Schwudke D., Sampaio J.L., Palm W., Riezman I., Dey G., Gupta G.D., Mayor S., Riezman H., Shevchenko A. (2010). Survival strategies of a sterol auxotroph. Development.

[9] Hobson R.P. (1935). On a fat-soluble growth factor required by blow-fly larvae: Distribution and properties. Biochem. J..

[10] Vanderplanck M., Moerman R., Rasmont P., Lognay G., Wathelet B., Wattiez R., Michez D. (2014). How does pollen chemistry impact development and feeding behaviour of polylectic bees?. PLoS ONE.

[11] Ferreira-Caliman M.J., da Silva C.I., Mateus S., Zucchi R., do Nascimento F.S. (2012). Neutral sterols of cephalic glands of stingless bees and their correlation with sterols from pollen. Psyche.

[12] Svoboda J.A., Thompson M.A., Herbert E.W., Shortino T.J., Szczepanik-Vanleeuwen P.A. (1982). Utilization and metabolism of dietary sterols in the honey bee and the yellow fever mosquito. Lipids.

[13] Svoboda J., Herbert E.W., Thompson M.J., Feldlaufer M.F. (1986). Selective sterol transfer in the honey bee: Its significance and relationship to other Hymenoptera. Lipids.

[14] Herbert E.W., Svoboda J.A., Thompson M.J., Shimanuki H. (1980). Sterol utilization in honey bees fed a synthetic diet: Effects on brood rearing. J. Insect Physiol..

[15] Feldlaufer M.F. (1986). Biosynthesis of makisterone A and 20-hydroxyecdysone from labeled sterols by the honey bee. Arch. Insect Biochem. Physiol..

[16] Chakrabarti P., Lucas H.M., Sagili R.R. (2020). Novel insights into dietary phytosterol utilization and its fate in honey bees (*Apis mellifera* L.). Molecules.

[17] Winston M.L. (1987). The Biology of the Honey Bee.

[18] Knecht D., Kaatz H.H. (1990). Patterns of larval food production by hypopharyngeal glands in adult worker honey bees. Apidologie.

[19] Crailsheim K., Schneider L.H.W., Hrassnigg N., Bühlmann G., Brosch U., Gmeinbauer R., Schöffmann B. (1992). Pollen consumption and utilization in worker honeybees (*Apis mellifera carnica*), dependence on individual age and function. J. Insect Physiol..

[20] Zheng A., Li J., Begna D., Fang Y., Feng M., Song F. (2011). Proteomic analysis of honeybee (*Apis mellifera* L.) pupae head development. PLoS ONE.

[21] Nie H., Liu X., Pan J., Li W., Li Z., Zhang S., Chen S., Miao X., Zheng N., Su S. (2017). Identification of genes related to high royal jelly production in the honey bee (*Apis mellifera*) using microarray analysis. Genet. Mol. Biol..

[22] Wu Y.Q., Zheng H.Q., Corona M., Pirk C., Meng F., Zheng Y.F., Hu F.L. (2017). Comparative transcriptome analysis on the synthesis pathway of honey bee (*Apis mellifera*) mandibular gland secretions. Sci. Rep..

[23] Wang K., Fan R.-L., Ji W.-N., Zhang W.-W., Chen X.-M., Wang S., Yin L., Gao F.-C., Chen G.-H., Ji T. (2018). Transcriptome analysis of newly emerged honeybees exposure to sublethal carbendazim during larval stage. Front. Genet..

[24] Zaluski R., Bittarello A.C., Vieira J.C.S., Braga C.P., Padilha P.D.M., da Silva Fernandes M., de Souza Bovi T., de Oliveira Orsi R. (2020). Modification of the head proteome of nurse honeybees (*Apis mellifera*) exposed to field-relevant doses of pesticides. Sci. Rep..

[25] Milone J.P., Chakrabarti P., Sagili R.R., Tarpy D.R. (2021). Colony-level pesticide exposure affects honey bee (*Apis mellifera* L.) royal jelly production and nutritional composition. Chemosphere.

[26] Troyer R.M., Ruby C.E., Goodall C.P., Yang L., Maier C.S., Albarqi H.A., Brady J.V., Bathke K., Taratula O., Mourich D. (2017). Exosomes from Osteosarcoma and normal osteoblast differ in proteomic cargo and immunomodulatory effects on T cells. Exp. Cell Res..

[27] Franceschini A., Szklarczyk D., Frankild S., Kuhn M., Simonovic M., Roth A., Lin J., Minguez P., Bork P., von Mering C. (2013). STRING v9.1: Protein-protein interaction networks, with increased coverage and integration. Nucleic Acids Res..

[28] Mi H., Poudel S., Muruganujan A., Casagrande J.T., Thomas P.D. (2016). PANTHER version 10: Expanded protein families and functions, and analysis tools. Nucleic Acids Res..

[29] Shannon P., Markiel A., Ozier O., Baliga N.S., Wang J.T., Ramage D., Amin N., Schwikowski B., Ideker T. (2003). Cytoscape: A software environment for integrated models of biomolecular interaction networks. Genome Res..

[30] Diz A.P., Carvajal-Rodríguez A., Skibinski D.O.F. (2011). Multiple hypothesis testing in proteomics: A strategy for experimental work. Mol. Cell. Proteomics.

[31] Kucharski R., Maleszka R.A. (1998). Royal jelly protein is expressed in a subset of kenyon cells in the mushroom bodies of the honey bee brain. Naturwissenschaften.

[32] Drapeau M.D., Albert S., Kucharski R., Prusko C., Maleszka R. (2006). Evolution of the yellow/major royal jelly protein family and the emergence of social behavior in honey bees. Genome Res..

[33] Buttstedt A., Moritz R.F.A., Erler S. (2014). Origin and function of the major royal jelly proteins of the honeybee (*Apis mellifera*) as members of the yellow gene family. Biol. Rev..

[34] Tian W., Li M., Guo H., Peng W., Xue X., Hu Y., Liu Y., Zhao Y., Fang X., Wang K. (2018). Architecture of the native major royal jelly protein 1 oligomer. Nat. Commun..

[35] Wyatt G.R., Davey K.G. (1996). Cellular and molecular actions of juvenile hormone. II. Roles of juvenile hormone in adult insects. Adv. Insect Physiol..

[36] Bellés X., Raikhel A. (2003). Vitellogenesis directed by juvenile hormone. Reproductive Biology of Invertebrates, Recent Progress in Vitellogenesis.

[37] Nelson C.M., Ihle K.E., Fondrk M.K., Page R.E., Amdam G.V. (2007). The gene vitellogenin has multiple coordinating effects on social organization. PLoS Biol..

[38] Amdam G.V., Fennern E., Havukainen H., Galizia C., Eisenhardt D., Giurfa M. (2012). Vitellogenin in honey bee behavior and lifespan. Honeybee Neurobiology and Behavior.

[39] Seehuus S.-C., Norberg K., Gimsa U., Krekling T., Amdam G.V. (2006). Reproductive protein protects functionally sterile honey bee workers from oxidative stress. Proc. Natl. Acad. Sci. USA.

[40] Weisiger R.A. (2002). Cytosolic fatty acid binding proteins catalyze two distinct steps in intracellular transport of their ligands. Mol. Cell. Biochem..

[41] Furuhashi M., Hotamisligil G.S. (2008). Fatty acid-binding proteins: Role in metabolic diseases and potential as drug targets. Nat. Rev. Drug Discov..

[42] Raychaudhuri S., Prinz W.A. (2010). The diverse functions of oxysterol-binding proteins. Annu. Rev. Cell Dev. Biol..

[43] Taylor A. (1993). Aminopeptidases: Structure and function. FASEB J..

[44] Jensen T.P., Buckby L.E., Empson R.M. (2004). Expression of plasma membrane Ca^2+^ ATPase family members and associated synaptic proteins in acute and cultured organotypic hippocampal slices from rat. Develop. Brain Res..

[45] Chakrabarti P., Rana S., Sarkar S., Smith B., Basu P. (2015). Pesticide-induced oxidative stress in laboratory and field populations of native honey bees along intensive agricultural landscapes in two Eastern Indian states. Apidologie.

[46] Henriksen J. (2006). Universal behavior of membranes with sterols. Biophys. J..

