# Supplementary figures and images for: Changes in Honey Bee Head Proteome in Response to Dietary 24-Methylenecholesterol
Source: Insects. 2020 Oct 29;11(11):743. doi: 10.3390/insects11110743 (PMC7693931; doi:10.3390/insects11110743)

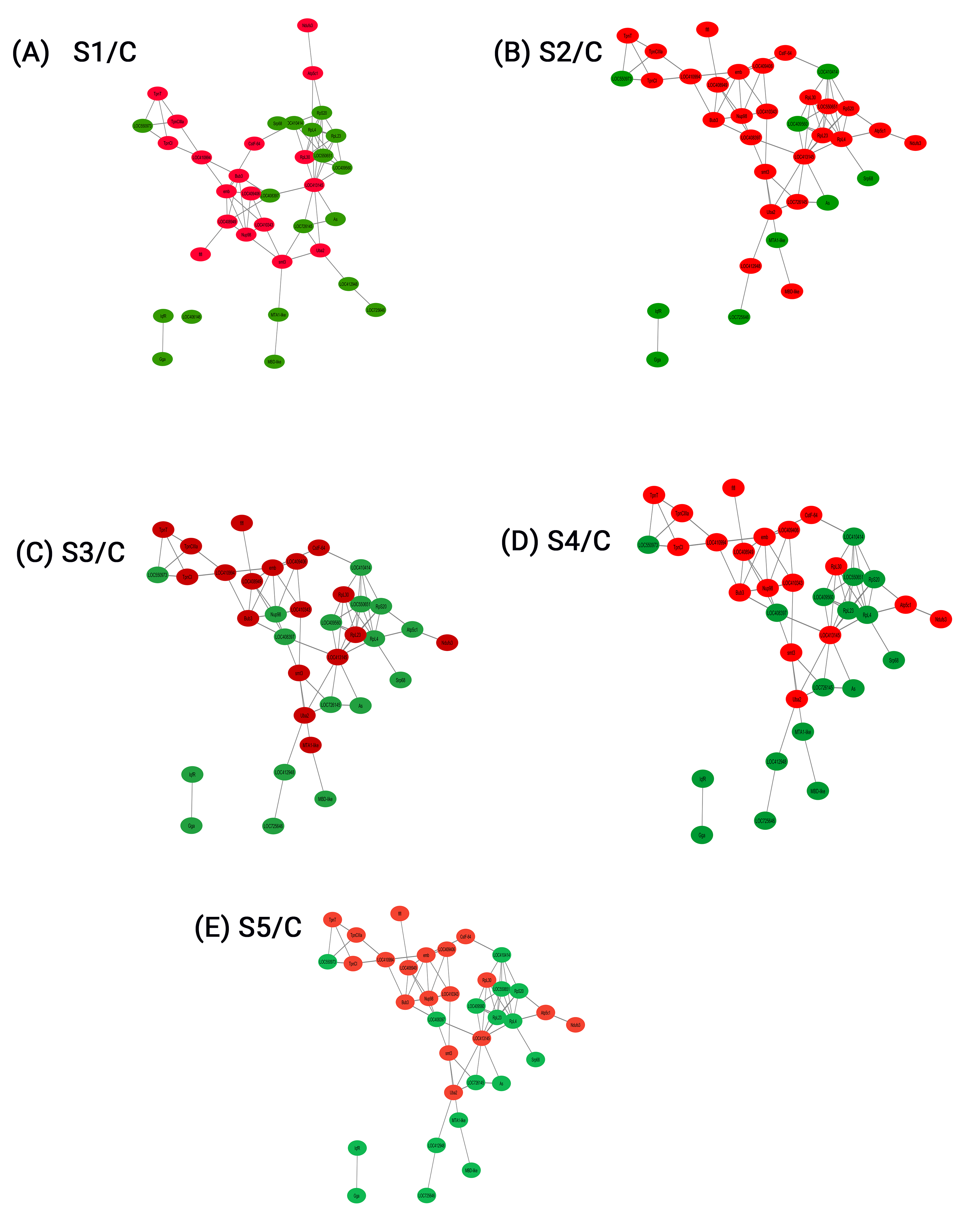

Supplement: Supplementary file 1 [file insects-11-00743-s001.zip › Chakrabarti and Sagili_supplementary information/Supplementary Figure 1.tif]
